# An unusual xylan in Arabidopsis primary cell walls is synthesised by GUX3, IRX9L, IRX10L and IRX14

**DOI:** 10.1111/tpj.12898

**Published:** 2015-06-04

**Authors:** Jenny C Mortimer, Nuno Faria-Blanc, Xiaolan Yu, Theodora Tryfona, Mathias Sorieul, Yao Z Ng, Zhinong Zhang, Katherine Stott, Nadine Anders, Paul Dupree

**Affiliations:** 1 Department of Biochemistry, University of CambridgeCambridge, CB2 1QW, UK; 2 Physical Biosciences Division, Lawrence Berkeley National Laboratory, Joint BioEnergy InstituteBerkeley, CA, 94720, USA

**Keywords:** Xylan, IRX10L, IRX9L, IRX14, GUX3, primary wall, *Arabidopsis thaliana*

## Abstract

Xylan is a crucial component of many plant primary and secondary cell walls. However, the structure and function of xylan in the dicotyledon primary cell wall is not well understood. Here, we characterized a xylan that is specific to tissues enriched in Arabidopsis primary cell walls. Unlike previously described xylans, this xylan carries a pentose linked 1–2 to the α-1,2-d-glucuronic acid (GlcA) side chains on the β-1,4-Xyl backbone. The frequent and precisely regular spacing of GlcA substitutions every six xylosyl residues along the backbone is also unlike that previously observed in secondary cell wall xylan. Molecular genetics, *in vitro* assays, and expression data suggest that IRX9L, IRX10L and IRX14 are required for xylan backbone synthesis in primary cell wall synthesising tissues. IRX9 and IRX10 are not involved in the primary cell wall xylan synthesis but are functionally exchangeable with IRX9L and IRX10L. GUX3 is the only glucuronyltransferase required for the addition of the GlcA decorations on the xylan. The differences in xylan structure in primary versus secondary cell walls might reflect the different roles in cross-linking and interaction with other cell wall components.

## Introduction

The plant cell wall is a complex, macromolecular structure that encapsulates every plant cell. It provides shape to individual cells, influencing their specific function, and providing structural integrity or plasticity to tissues and organs as needed. Cells that are undergoing expansion are surrounded by a thin, pectin rich primary cell wall. Once expansion is completed, a thicker secondary cell wall is laid down on the inside of the primary wall, chiefly to provide strength. Completion of secondary cell wall deposition is accompanied by lignification, and often the cell enters programmed cell death (Buchanan *et al*., 2000).

According to a current model of the plant cell wall, the primary wall consists of long, rigid cellulose microfibrils embedded in a highly hydrated matrix of non-cellulosic polysaccharides and glycoproteins (Cosgrove, 2005). In the majority of higher plants (including Arabidopsis) this non-cellulosic matrix is formed mostly of xyloglucan and pectins. Cellulose:xyloglucan interactions are thought to influence cell expansion (Cosgrove, 2014). Small amounts of xylan have been reported in primary cell walls (Darvill *et al*., 1980; Zablackis *et al*., 1995; Hervé *et al*., 2009), but any function for this hemicellulose is unclear. In the Poales (e.g. grass) primary cell wall, glucuronoarabinoxylan is the dominant hemicellulose, suggesting that some hemicellulose replacement has taken place during the evolution of plants (Carpita, 1996). The secondary cell wall also consists of a framework of cellulose microfibrils, and these are also associated with xylan and glucomannan hemicelluloses, over which lignin, a complex phenolic network, is deposited (Scheller and Ulvskov, 2010). Xylan is an essential component of these secondary cell walls, since Arabidopsis xylan biosynthesis mutants have collapsed xylem vessels and if there is a complete loss of xylan biosynthesis, the plants are not viable (Wu *et al*., 2009).

Xylan polysaccharides consist of a backbone of β-1,4-linked-d-xylopyranosyl (Xyl) residues with a degree of polymerisation (DP) that is estimated to range from ∼150 xyloses (X_150_) in Arabidopsis secondary cell walls to ∼X_1000–4000_ in wheat endosperm (Freeman *et al*., 2015). In Arabidopsis, several different glycosyltransferases (GTs) have been identified as having a role in secondary cell wall xylan backbone synthesis, including multiple members of GT43 (IRX9 and IRX9L; IRX14 and IRX14L) and GT47 (IRX10 and IRX10L) (Brown *et al*., 2007, 2009; Pena *et al*., 2007; Persson *et al*., 2007; Wu *et al*., 2009). It is unclear to what extent the similar proteins in these three pairs are functionally specialised. Wu *et al*. (2009) suggested that for secondary cell wall xylan, these pairs are functionally interchangeable, as the *irx9, irx14* and *irx10* phenotypes can be complemented by overexpression of IRX9L, IRX14L and IRX10L respectively.

The xylan backbone is variously substituted with α-1,2-d-glucuronic acid (GlcA), 4-*O*-methyl-α-1,2-d-glucuronic acid (MeGlcA), α-1,2- or α-1,3-l-arabinofuranosyl (Ara*f*) residues, as well as with *O*-acetyl (Ac) groups, depending on the species, tissue and age. Dicot secondary cell wall xylan is substituted solely by acetate and [Me]GlcA (Scheller and Ulvskov, 2010). It has been hypothesised that the GlcA methylation may influence cross-linking of the xylan to the lignin, and indeed loss of xylan methylation led to a slight increase in xylan accessibility (Urbanowicz *et al*., 2012). Eucalypts appear to have an unusual xylan structure with α-1,2-d-Gal linked to the GlcA branches (Shatalov *et al*., 1999). In contrast, essentially nothing is known about dicot primary cell wall xylan structure. In grasses, primary and secondary cell wall xylan α-1–3-Ara substitutions can be substituted with ferulate esters (Fer), which can then be further decorated with β-1,2-Xyl (Wende and Fry, 1997) and β-1–4-l-Gal (Saulnier *et al*., 1995), although the abundance of these different side decorations is species and tissue dependent. These feruloylated Ara residues enable xylan–xylan and xylan–lignin cross-linking (Grabber, 2005).

Identification of the GTs responsible for the addition of the GlcA to Arabidopsis secondary cell wall glucuronoxylan, the family GT8 proteins GUX1 and GUX2 (Mortimer *et al*., 2010; Rennie *et al*., 2012), has enabled us to test the role of sugar substitutions. The absence of secondary wall xylan GlcA had no impact on Arabidopsis growth, and a relatively minor effect on stem strength (Mortimer *et al*., 2010). However, detailed characterization revealed strikingly different patterns of GlcA substitution of the xylan (Bromley *et al*., 2013). A ‘major’ domain is decorated by GUX1 where substitutions are widely spaced but an even number of Xyl residues apart. A ‘minor’ domain is decorated by GUX2 in which substitutions are tightly clustered but have no preference for odd or even spacing. Precise patterning has also been detected in xylan acetylation (Busse-Wicher *et al*., 2014). Molecular dynamic simulations have led to a proposal that the different domains are able to make different non-covalent interactions between the xylan and the cellulose fibrils (Busse-Wicher *et al*., 2014). Even spacing between acetate and [Me]GlcA decorations allows xylan to fold with a two-fold helical screw. This fold is compatible with hydrogen bonding of xylan with glucan chains in cellulose.

There are very limited biochemical data concerning any xylan molecules in Arabidopsis primary cell walls, yet discovery of the structure and biosynthetic mechanism of primary cell wall xylan is essential for understanding its function. Here, we present a previously uncharacterized primary wall xylan with an unusual structure and describe its biosynthetic machinery.

## Results

### A xylan with unusual side chains is present in primary cell wall rich tissues

**Figure 1:**
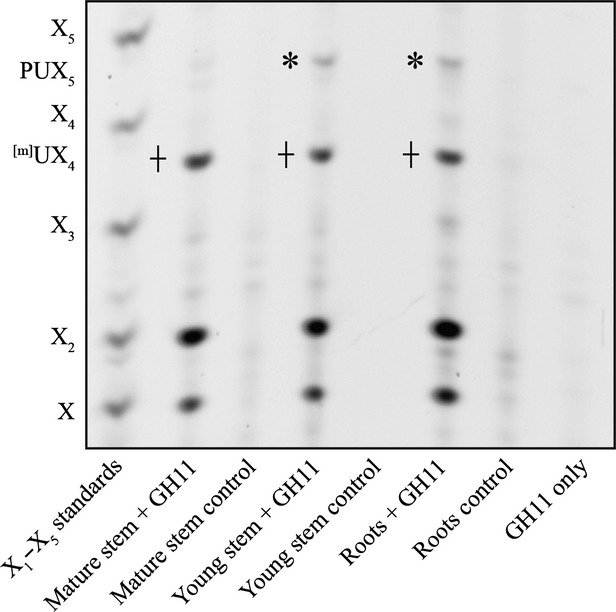
Xylan structure in primary cell wall rich tissues. PACE analysis of xylanase (GH11)-digested AIR from mature stem, young stem and roots and respective no-enzyme controls. Xylo-oligosaccharide standard: X_1_–X_5_, GH11 only: enzyme only control. Note: PUX_5_ structure, marked with *, is not present in mature stem, whereas ^[m]^UX_4_, marked with +, is present in all tissues analysed.

Little information is known about the structure of xylan in the primary cell wall of dicotyledons. Therefore, we analysed the structure of xylan in Arabidopsis tissues in which the primary cell wall is highly abundant. First, alcohol insoluble residues (AIR) were produced from young stems and roots, digested with the GH11 xylanase (EC 3.2.1.8) and the xylan structure analysed by polysaccharide analysis by carbohydrate gel electrophoresis (PACE) (Figure1 and see Figure S1a for explanation of xylanolytic hydrolase specificities). Previous work had revealed that xylan from secondary wall rich tissues of various different dicotyledons (including Arabidopsis, tobacco, willow) hydrolysed with this xylanase releases the following oligosaccharides: xylose (X), xylobiose (X_2_), and aldopentouronic acid with (^m^UX4) or without (UX_4_) a 4-*O*-methyl group on the glucuronic acid (Mortimer *et al*., 2010; Yamaguchi *et al*., 2010; Wan *et al*., 2014). These two oligosaccharides do not separate under the PACE conditions used in this study, and are together referred to here as ^[m]^UX_4_. The primary cell wall rich tissues tested had a different xylan structure compared to tissue rich in secondary walls (mature stems) (Figure1). Specifically, an oligosaccharide was identified that migrated between X_4_ and X_5_ on the PACE gel, labelled here as PUX_5_. AIR was produced from liquid-grown root-derived callus, and analysed as above (Figure S1b). Xylanase-dependent bands X, X_2_, ^[m]^UX_4_ and PUX_5_ were again detected, but the quantity of xylan was extremely small.

### MALDI collision induced dissociation (MALDI-CID) and nuclear magnetic resonance (NMR) analysis shows that the PUX_5_ structure possesses a side chain of an pentose linked 1–2 to α-1,2-GlcA

**Figure 2:**
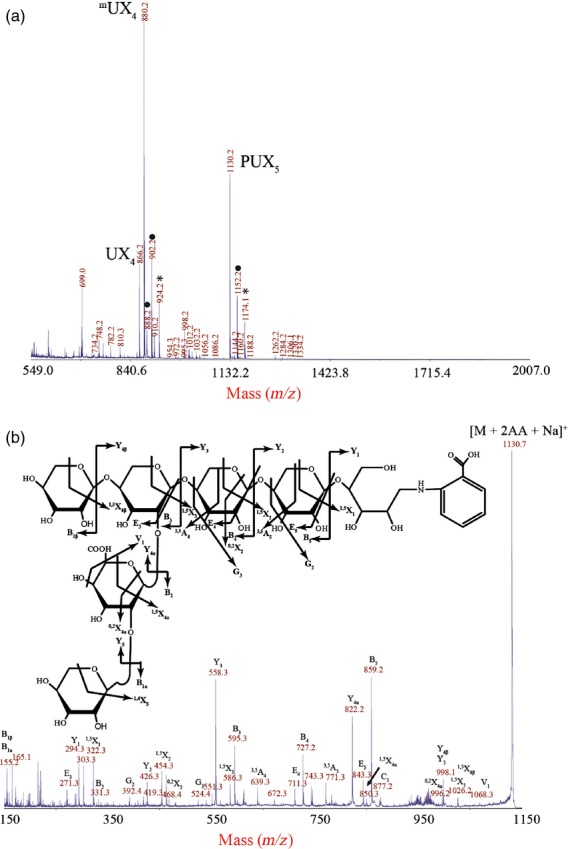
Structural analysis of the PUX_5_ oligosaccharide. (a) Xylanase (GH11)-digested and 2-AA labelled AIR from young WT stem analysed by MALDI-ToF-MS to generate a series of sodiated adducts [M + Na]^+^. Peaks with dots are doubly sodiated. Peaks with stars are trebly sodiated. The peak corresponding to PUX_5_ with mass GlcAPent_6_ (*m/z* 1130.2) was selected for structural analysis using high intensity MALDI-CID. (b) The CID spectrum identifies PUX_5_ as 1,2-pentose-α-1,2-GlcA on a backbone of five β-1,4-Xyl.

In order to characterize the structure of the xylan further, GH11 digested AIR from young stems was reductively aminated with the fluorophore 2-AA, and analysed by MALDI-ToF-MS. In addition to ^m^UX4 and UX_4_, a primary wall specific peak corresponding to PUX_5_ was identified_,_ with *m/z* 1130.2 suggesting it consists of six pentoses and one uronic acid (GlcA) (Figure2a). Unlike xylan previously characterized from secondary wall rich tissues (Mortimer *et al*., 2010), the unmethylated form of GlcA was dominant. PUX_5_ was selected for CID in order to derive the structure (Figure2b). A backbone consisting of five 1,4-linked Xyl residues was indicated by the series of Y_1–4β_ and ^1,5^X_1–4β_ ions (Domon and Costello, 1988). Furthermore, the 440 Da mass difference between the Y_3_ and Y_4β_ ions suggests that the penultimate Xyl from the reducing end on the pentasaccharide xylan backbone is modified with a uronic acid (176 Da) and a pentose (132 Da) residue (hence pentosyl-UX_5_, PUX_5_). The presence of the Y_4α_ and Υ_5_ ions (*m/z* 822.2 and 998.1, respectively) indicates that the uronic acid is linked to the xylan backbone pentasaccharide, and a terminal pentose residue is linked to the uronic acid. The presence of the E_3_ elimination ion (Chai *et al*., 2001) (*m/z* 271.3) indicates that the uronic acid is linked at the O-2 of the Xyl residue as expected of GlcA decorations on xylan. The ^0,2^X_4α_ cross-ring fragment (*m/z* 996.1) suggests that the terminal pentose is 1,2-linked to the uronic acid. It was not possible to determine the identity of the 1,2-linked pentose due to poor fragmentation of the non-reducing end of the molecule.

In support of this structural assignment, the xylan was hydrolysed with a xylanase from the GH30 family (EC 3.2.1.136), which cuts leaving uronic acid on the −2 position, giving a ladder of oligosaccharides which carry a single uronic acid (Bromley *et al*., 2013; Figure S1a). MALDI-CID of a peak with *m/z* 1141 (equivalent to seven pentoses and a uronic acid) identified an oligosaccharide which consisted of a β-1,4-linked Xyl backbone with a uronic acid carrying a 2-linked pentose (Figure S2a,d).

**Figure 3:**
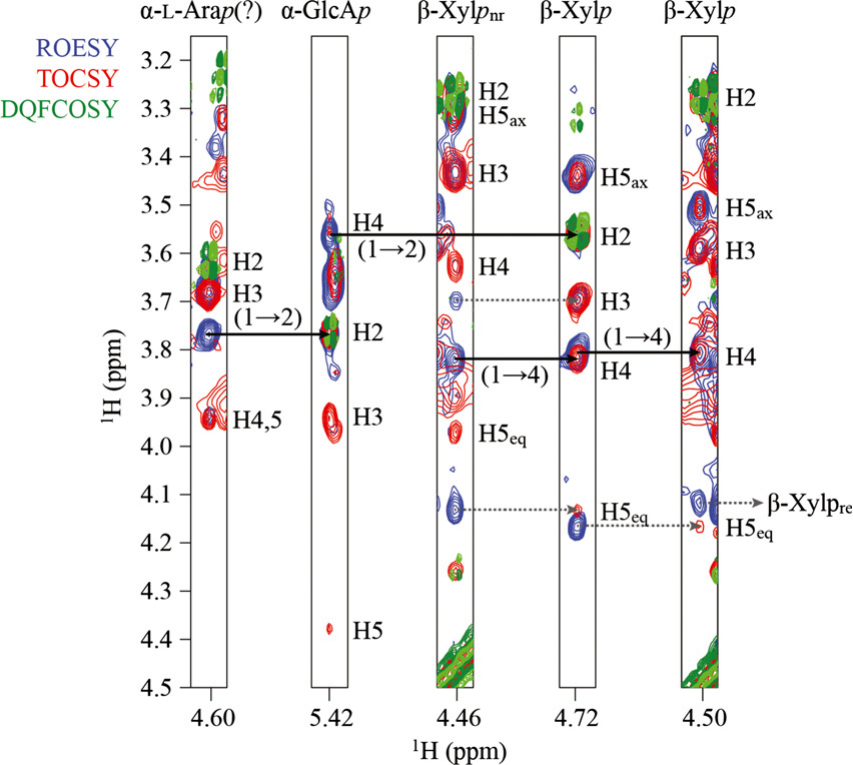
Nuclear magnetic resonance (NMR) analysis of PUX_5_. H-1 strip plots from 2D ^1^H-^1^H TOCSY (red) ROESY (blue) and DQFCOSY (green) spectra, showing the nuclear Overhauser effect (NOE) connectivity arising from the pentose-α-1,2-Glc*p*A-α-1,2-Xyl*p* and β-Xyl*p*-β-1,4-Xyl*p* glycosidic linkages.

For analysis by solution-state NMR, young stem AIR was exhaustively digested with GH11 xylanase and α-glucuronidase, separated by size exclusion chromatography (SEC), and fractions rich in PUX_5_ (as determined by PACE) were collected and pooled (Figure3). ^1^H chemical-shift assignments were primarily obtained using 2D ^1^H–^1^H total correlation spectroscopy (TOCSY) and rotating frame Overhauser effect spectroscopy (ROESY). The H-1/H-2 peaks in a double quantum filtered correlation spectroscopy (DQFCOSY) were used to remove ambiguities in the assignments of H-2. ^13^C assignments were obtained using ^13^C HSQC and H2BC experiments (although the latter was incomplete due to the low concentration of the sample). The non-reducing end Xyl*p* residue of the backbone was readily identified from H-1/C-1 chemical shifts and the two internal Xyl*p* were assigned using the intense (1→4) linkages apparent in the ROESY spectrum (Figure3). The H-2 and C-2 of the Xyl*p* adjacent to the non-reducing end were downfield-shifted, characteristic of a glycosidic link, and the (1→2) linkage from α-Glc*p*A was apparent from an intense rotating frame Overhauser effect (ROE) (Figure3). The H-2 and C-2 of the α-Glc*p*A were also downfield shifted and could be linked to a pentose residue via an intense ROE peak. The chemical shifts of the pentose were possibly consistent with α-l-arabinopyranose (Ara*p*) and the low intensity of the H-1/H-4 TOCSY peak were consistent with an equatorial H-4 (e.g. α-l-Ara*p* or β-d-Xyl*p*), although this could not be assigned with certainty. The shifts were inconsistent with α-l-Ara*f*. Peaks arising from the reducing end β-Xyl*p* were relatively weak and appeared in a region of the spectrum that was heavily overlapped, however they were tentatively assigned using an ROE between the H-5_eq_ and the H-1 of the preceding residue. Chemical-shift assignments are shown in Table S1. Taken together the NMR analysis identifies the PUX_5_ branch structure as α-1,2-l-Ara*p*-α-1,2-GlcA or β-1,2-d-Xyl*p*-α-1,2-GlcA. The branch structure was resistant to β-xylosidase, β-l-arabinopyranosidase and α-l-arabinofuranosidase, supporting the assignment of the pentose as the unusual sugar α-1,2-l-Ara*p*.

### The GUX3 glycosyltransferase is responsible for addition of the GlcA in the PUX_5_ structure

**Figure 4:**
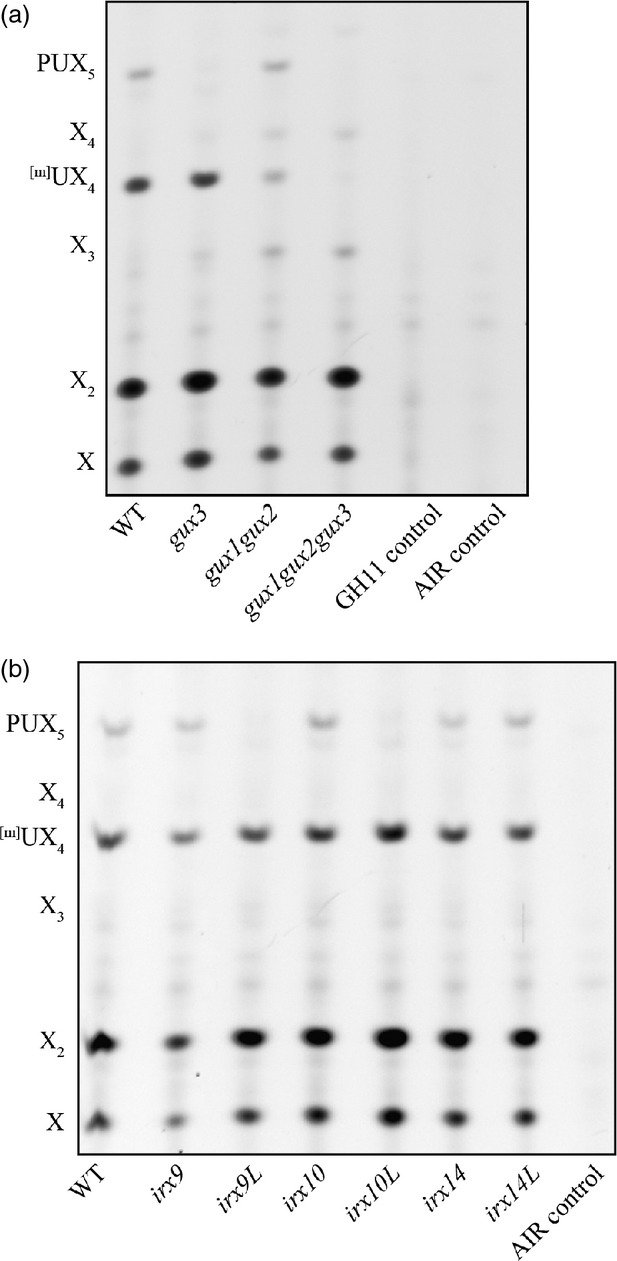
The PUX_5_ structure is dependent on GUX3, IRX9L, and IRX14/IRX14L. PACE analysis of xylanase (GH11)-digested AIR from young stem of (a) *gux* mutants, and (b) *irx* mutants, hydrolysed with GH11 and analysed by PACE, alongside controls. Xylo-oligosaccharide standard: X_1_–X_4_ positions are shown.

Members of the GT8 glycosyltransferases were shown to act as glucuronyltransferases on xylan (Mortimer *et al*., 2010; Rennie *et al*., 2012). In order to identify the enzyme adding the GlcA to the PUX_5_ structure, we analysed the xylan structure in T-DNA knock-out lines of all 5 GUX family members of Arabidopsis. We used the previously published knock-out lines for *gux1* and *gux2* (Mortimer *et al*., 2010), and we isolated transcriptionally null homozygous T-DNA lines for *GUX3* (*gux3-1, gux3-2*) and for *GUX4* and *GUX5* (*gux4-1* and *gux5-1*). Since GUX4 and GUX5 show 70% protein identity and may be redundant, we also generated the double mutant (*gux4gux5*) (Figure S3). In growth, all mutants were indistinguishable phenotypically from WT. AIR was prepared from young stems and roots of the *gux* mutant lines, hydrolysed with a xylanase and analysed by PACE, as described above (Figures4a and S4). Compared with WT, both *gux3* lines had undetectable quantities of PUX_5_ oligosaccharide, but only a slight reduction in the ^[m]^UX_4_. In contrast, the *gux1gux2* double mutant showed unaltered abundances of PUX_5_, but strongly reduced ^[m]^UX_4_. *gux4gux5* showed no alteration to xylan structure, suggesting that in the tissues analysed, they played no significant role in xylan decoration. This finding suggests that GUX3 is solely responsible for [Me]GlcA substitution of the primary wall-specific xylan.

A triple mutant *gux1gux2gux3* showed no detectable [Me]GlcA decoration in roots or young stems, implying that these three proteins are the major xylan glucuronosyltransferases. Unlike a previous report (Lee *et al*., 2012), we did not observe any gross phenotypical differences or an *irx* phenotype in the *gux1gux2gux3* triple mutant (Figure S5).

### The GUX3-dependent xylan has a distinct pattern of GlcA substitution

Recently, our group demonstrated that the [Me]GlcA substitutions of secondary wall xylan show two distinct patterns in discrete domains (Bromley *et al*., 2013). The major domain, for which GUX1 is responsible, has large, even numbers of Xyl residues between each [Me]GlcA decoration. The minor domain, which is decorated by GUX2 has densely packed [Me]GlcA substitutions on both even and odd residues (Bromley *et al*., 2013). The spacing was determined using a GH30 xylanase (as described above), which strictly requires a [Me]GlcA substitution in the −2 position with respect to the cleavage site.

**Figure 5:**
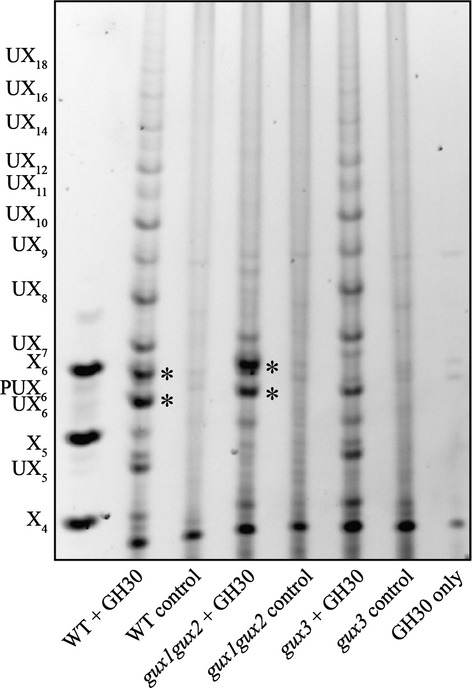
GlcA spacing on primary wall xylan. PACE analysis of xylanase GH30-digested AIR from WT, *gux1gux2* and *gux3* roots. The GUX3-dependent oligosaccharides are marked with *.

To understand whether the primary cell wall xylan showed a [Me]GlcA spacing pattern as characterized for xylan in the secondary cell wall, we isolated AIR from WT, *gux3* and *gux1gux2* roots, hydrolysed it with GH30 and analysed the xylan structure by PACE (Figure5). In the WT root fingerprint, it was evident that there were two abundant oligosaccharides (marked with asterisks), as compared with the fingerprint previously reported for WT mature stem (Bromley *et al*., 2013). One of these oligosaccharides migrated with UX_6_ and the other migrated a little more slowly. The *gux3* mutant specifically lacked these two oligosaccharides. In contrast, the *gux1gux2* mutant only produced these oligosaccharides. This finding suggests that some of the root xylan shows a unique GUX3-dependent spacing of its [Me]GlcA decorations, in which the every sixth Xyl carries a sugar substitution. The remaining oligosaccharides in *gux3* showed a pattern very similar to WT mature stems. This implies that the roots also possess a xylan that has a GUX1/GUX2-dependent [Me]GlcA distribution akin to that of the secondary cell wall rich tissues. This is likely the xylan derived from the root cells that undergo secondary cell wall deposition, mainly the vasculature. The GUX3 and GUX1/GUX2–dependent xylan [Me]GlcA patterns were apparently independent and additive.

WT, *gux3* and *gux1gux2* root AIR hydrolysed with GH30 was also analysed by MALDI-TOF MS (Figure S2). Consistent with the PACE data, the *gux3* roots produced a ladder of oligosaccharides, but noticeably lacked the Pent_7_GlcA (*m/z* 1141; Figure S2b) oligosaccharide of six backbone Xyl residues carrying a pentose on the GlcA (PUX_6_, Figure S2d). The *gux1gux2* roots by comparison only had two major peaks (*m/z* 1023 and 1141; Figure2c), corresponding to UX_6_ and PUX_6_ (Figure5). As before, the oligosaccharide product pattern analysed by MALDI from *gux1gux2* and *gux3* was additive to that of the WT, showing that the different GUX enzymes act on different xylan polymers or different areas of a xylan polymer, thus creating their unique [Me]GlcA substitution pattern. A proposed structure of this xylan is shown in Figure S1(c).

### Cells actively synthesising the primary cell wall express a subset of xylan synthesis genes

In order to understand whether a specific set of enzymes synthesise the xylan backbone decorated by GUX3, we analysed the transcriptome of Arabidopsis root callus culture. In contrast with cell wall from young stem and roots, where, in addition to primary cell walls, secondary cell wall is produced in e.g. the stele of the root and the vasculature of the hypocotyl, secondary cell wall production in callus culture is absent. This view is supported by the pattern of cellulose synthase (*CESA*) gene expression observed: the primary wall CESAs are highly expressed (particularly *CESA1, CESA3* and *CESA6*) whereas the secondary wall *CESA* expression is essentially undetectable. Similar CESA expression results were obtained by mining a proteomics data set derived from the same callus lines (Table 1; Nikolovski *et al*., 2012). Interestingly, the xylan biosynthetic genes *IRX9L, IRX10L* and *IRX14,* as well as *GUX3* are highly expressed in callus (Table 1 and Data S1). *IRX10* had an intermediate level of expression. In contrast, *IRX9, IRX14L, GUX1* and *GUX2* had extremely low levels of expression, close to undetectable. Proteomic analysis of membrane proteins in the same callus (Nikolovski *et al*., 2012) was consistent with this result, with unique peptides identified only from IRX10L, IRX14 and GUX3. No peptides were detected for any of the other xylan biosynthetic proteins. These data are also consistent with analysis of publicly available root development microarray data (Brady *et al*., 2007) which revealed that very young, still expanding root cells showed a similar profile of xylan GT expression to the callus (Figure S6). In contrast, older, fully expanded root tissue, particularly the stele, which contains the vasculature, has a xylan-related GT profile more similar to that seen for mature stems (Figure S6). Taken together, there is a primary wall specific pattern of expression for xylan-related biosynthetic genes, namely *IRX9L, IRX10L, IRX14* and *GUX3*.

**Table 1:** Transcriptomic and proteomic data for selected cell wall synthesis genes and their homologues

AGI	Gene name	RMA computed expression value (Log_2_)	# Peptides
Primary wall cellulose synthases			
AT4G32410	CESA1	10.569	15
AT5G05170	CESA3	11.942	16
AT5G64740	CESA6	10.526	ND
AT4G39350	CESA2	8.255	ND
AT2G21770	CESA9	4.982	ND
Secondary wall cellulose synthases			
AT5G17420	CESA7/IRX3	5.546	ND
AT4G18780	CESA8/IRX1	2.597	ND
AT5G44030	CESA4/IRX5	2.037	ND
Xyloglucan biosynthetic enzymes			
AT3G28180	CSLC4	9.963	6
AT3G62720	XXT1	10.794	6
AT4G02500	XXT2	9.656	4
AT1G74380	XXT5	9.689	10
Xylan reducing end oligosaccharide biosynthetic enzymes			
AT5G54690	IRX8	5.249	ND
AT2G28110	IRX7/FRA8	7.959	ND
AT1G19300	PARVUS	5.615	ND
Xylan biosynthetic enzymes			
AT4G36890	IRX14	8.494	3
AT1G77130	GUX3	8.760	6
AT5G61840	IRX10L	9.516	11
AT1G27600	IRX9L	9.396	ND
AT1G27440	IRX10	6.921	ND
AT2G37090	IRX9	3.801	ND
AT5G67230	IRX14L	2.128	ND
AT3G18660	GUX1	1.997	ND
AT4G33330	GUX2	1.972	ND

RNA and proteins were extracted from WT Col0 liquid grown callus. Full transcriptomic data can be found in Data S1. RMA values less than about 6 can be considered unreliable, since their low expression results in a low signal to noise ratio, but they have been included for completeness. Proteomic data were extracted from Nikolovski *et al*. (2012).

### Callus microsomes exhibit xylan xylosyltransferase and glucuronosyltransferase activity and can synthesise xylan

**Figure 6:**
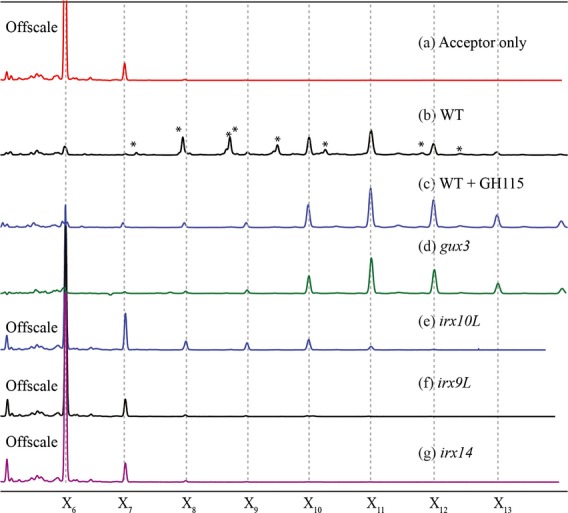
XylT and GlcAT activity in callus culture microsomes.An acceptor (APTS-labelled X_6_) and substrates (UDP-Xyl, UDP-GlcA) were incubated for 5 h in the absence (a) or presence (b–g) of callus microsomes, and analysed by capillary electrophoresis (CE). WT Col0 callus gave a ladder of products (b) which following hydrolysis with GH115 α-glucuronidase resulted in a ladder of xylo-oligosaccharides (c). Peaks marked with an * are GH115 sensitive. *gux3* callus (d) gave a very similar trace to WT + GH115 implying that little or no GlcA addition occurred. *irx10L* (e) *irx9L* (f) and *irx14* (g) showed little or no extension of the acceptor implying that xylan synthesis was essentially absent. The acceptor (X_6_) peak has been cropped in all traces except (b–d) for clarity.

Previous work has shown that cultured cells possess primary walls with a heteroxylan component (Darvill *et al*., 1980; Popper and Fry, 2005). To test whether our Arabidopsis callus culture is a suitable model for primary cell wall xylan biosynthesis, xylosyltransferase (XylT) and glucuronyltransferase (GuxT) activities were studied. Microsomes were incubated in the presence of an acceptor [xylohexaose (X_6_) labelled with the fluorophore APTS at its reducing end] and UDP-Xyl and UDP-GlcA as the substrates (Figure6). After 5 h, essentially all acceptor had been consumed, and many longer oligosaccharides were detected (Figure6a,b). To determine whether any of the assay products carried α-GlcA, they were then incubated with a family GH115 xylan-specific α-glucuronidase (Figure6c). This enzyme is capable of hydrolysing [Me]GlcAs at any position along the xylan backbone (Rogowski *et al*., 2014). This revealed that a range of xylan oligosaccharides had been synthesised, up to and exceeding DP14, and that the majority of these carried at least one GlcA substitution (Figure6). Therefore, callus-derived microsomes were capable of synthesising glucuronoxylan. Minor peaks, which did not co-migrate with unsubstituted xylo-oligosaccharides and which may include the PUX_5_ oligosaccharide, remained following GH115 digestion.

### IRX9L, IRX10L, IRX14 and GUX3 are required for xylan synthesis by callus microsomes

In order to investigate the activity of the subset of xylan biosynthesis GTs expressed in callus culture, we generated callus from *irx9L, irx10L, irx14* and *gux3* mutants, and then performed XylT/GuxT assays (Figure6d–g). *gux3* callus-derived microsomes produced a ladder of unsubstituted β-1,4-Xyl oligosaccharides with DP 7-14, with DP 11-14 being the most abundant (Figure6d). Longer DP oligosaccharides may be synthesised in the assays, but these were difficult to detect likely due to the decreasing solubility of longer unsubstituted xylan oligosaccharides. The absence of GlcA decorations in *gux3* assay products indicates that GUX3 is responsible for essentially all GlcA substitutions in primary cell wall xylan of callus. It also shows that, similar to the mechanism of secondary cell wall xylan formation (Mortimer *et al*., 2010), XylT activity is able to proceed unhindered in the absence of GlcAT activity.

Both *irx9L* and *irx14* membranes lacked detectable XylT activity (Figure6f,g). Membranes from *irx10L* callus had a small amount of residual XylT activity, but it was extremely low compared to WT (Figure6e). After 5 h of incubation, in *irx10L* the majority of the acceptor remained unextended and a minor fraction was extended by one to four Xyl. In contrast, in WT or *gux3* the acceptor had been entirely consumed and longer oligosaccharides had been produced. These data show that IRX9L, IRX10L and IRX14 are required for primary cell wall xylan biosynthesis in callus, and that GUX3 is the GlcAT for this xylan.

### The subset of XylTs, IRX9L, IRX10L and IRX14 synthesise the primary cell wall xylan

The role of the GTs implicated *in vitro* in the primary wall xylan backbone synthesis was studied further young stems, tissues which are rich in primary cell wall but which are also developing some vascular tissues with secondary cell walls. We investigated the presence of the primary cell wall specific PUX_5_ oligosaccharide in xylan synthesised in young stems of *irx9, irx9L, irx10, irx10L, irx14* and *irx14L* (Figure4b). Consistent with the expression and activity data gained from callus culture, PUX_5_ was undetectable in the xylan from *irx9L* and *irx10L* mutants. In *irx14,* PUX_5_ was present but clearly reduced. However, *irx9, irx10, irx14L,* although having the expected general decrease in secondary cell wall xylan backbone quantity, showed unchanged quantities of the PUX_5_ oligosaccharide. This supports the view that the PUX_5_ modified xylan is a primary cell wall xylan, and is synthesised specifically by IRX9L, IRX10L and IRX14. IRX14L may be partially redundant to IRX14 in the young stem primary cell wall xylan synthesis.

### Promoter exchanges suggest that IRX9 and IRX10 proteins can functionally replace IRX9L and IRX10L in primary cell wall xylan synthesis

**Figure 7:**
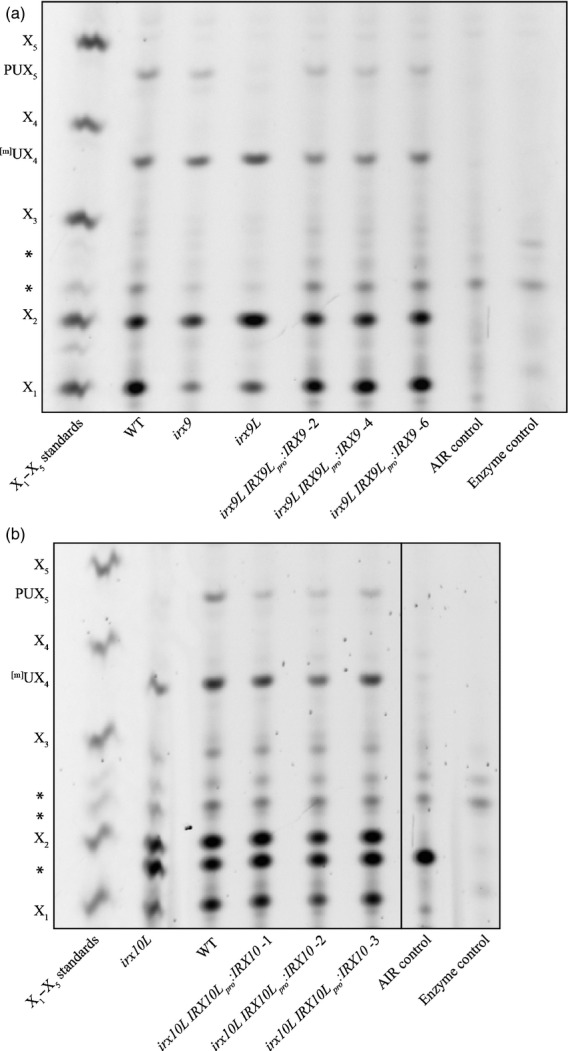
Complementation of *irx9L* and *irx10L* xylan phenotype with IRX9 and IRX10 respectively.PACE analysis of xylanase (GH11)-digested AIR from (a) *irx9L* young stems expressing IRX9 under the control of the IRX9L promoter (*IRX9L*_*pro*_*:IRX9*) and (b) *irx10L* young stems expressing IRX10 under the control of the IRX10L promoter (*IRX10L*_*pro*_*:IRX10*). Some lanes have been excised for clarity, as shown by the black line. AIR was prepared from young inflorescent stems, hydrolysed with xylanase GH11 and analysed by PACE. Three independently transformed lines are shown. Background bands are indicated by an *.

We considered whether the requirement specifically for IRX9L and IRX10L might arise because these are specialised for primary wall xylan synthesis, and they might be functionally distinct from IRX9 and IRX10 that act uniquely in secondary cell wall synthesis. Alternatively, IRX9 and IRX10 may be functionally identical to the primary cell wall xylan biosynthesis enzymes but unable to compensate for IRX9L and IRX10L because they are not expressed in the necessary tissues. A promoter exchange experiment was designed in which *IRX9* was cloned under the *IRX9L* promoter and transformed into the *irx9L* mutant. Three independently transformed lines were isolated, AIR was prepared from young stems of the mono-insertional and homozygous T3 generation, digested with the GH11 xylanase and analysed by PACE (Figure7a). In the *pIRX9L:IRX9* lines the PUX_5_ oligosaccharide was once again present. Similar results were obtained with *pIRX10L:IRX10* lines (Figure7b). Therefore, IRX9 and IRX10 are able to replace the function of IRX9L and IRX10L in primary cell wall xylan biosynthesis.

## Discussion

Xylan is the second most abundant biopolymer on the planet, and yet despite this situation, we still do not understand how it is synthesised at the biochemical level. In particular, it is not clear why so many different GTs have been implicated in xylan synthesis and how they may work together to produce different xylan structures. Moreover, the function of different types of xylans and how they interact with other cell wall components is largely unknown. Here, we have characterized a previously unreported type of xylan and shown that GUX3 is specifically responsible for adding the GlcA side chains. We have shown that a subset of the known genes responsible for backbone elongation, IRX9L, IRX10L and IRX14 synthesise this xylan.

What makes the xylan we describe in this work different to secondary cell wall xylan? First, this xylan is typified by GlcA substitution carrying a pentose decoration. The pentose is linked 1–2 to the α-1,2-GlcA branches. Such a structure has not been reported elsewhere to our knowledge, and it is not present in Arabidopsis secondary cell wall xylan. Second, the GUX3 glucuronyltransferase is responsible for GlcA side chain addition, whereas in the secondary cell wall GUX1 and GUX2 are required. Third, the resulting pattern of GlcA substitution is different from that reported in dicot secondary cell walls in that the distance of GlcA substitutions along the Xyl backbone is fixed at every sixth Xyl residue. Fourth, the GlcA remains largely unmethylated. Last, a non-redundant subset of xylan backbone synthesis GTs, those that are expressed in primary cell wall synthesising cells, are required for the synthesis of this xylan.

This unusual xylan is most abundant in some primary cell wall rich organs, particularly roots and young stem of Arabidopsis. However, secondary cell wall is present in these organs, so we cannot exclude that the xylan structure is in the secondary as well as primary cell walls in roots and young stems. Nevertheless we believe it likely that we are looking at a xylan type specific for the primary cell wall. This xylan is different in its structure from the xylan described for the secondary cell wall in Arabidopsis mature stem. Additionally, digestion of WT and mutant xylan with the GH30 glucuronoxylanase showed that the GlcA substitutions are clustered together on xylan at every sixth residue, independent of the substitution activities of GUX1 and GUX2 that act on secondary cell wall xylan. Third, the subset of xylan backbone biosynthetic GTs expressed in callus culture, where secondary cell wall formation is not described, are responsible for the synthesis of this type of xylan in roots and young stems.

It has been hypothesised that the gene duplication of xylan-related GTs has resulted in a ‘major’ and ‘minor’ set of XylTs in secondary cell wall xylan synthesis (Wu *et al*., 2010). Here we describe that a subset, and not all, of the *irx* genes synthesise the primary cell wall xylan, suggesting that rather than a minor and major function, IRX9L, IRX10L and IRX14 act in the synthesis of the primary cell wall xylan, whereas IRX9, IRX10 and IRX14L predominantly synthesise secondary cell wall xylan. In secondary cell wall xylan synthesis there is some functional redundancy between the pairs of proteins, as double *irx* mutants in these pairs of enzymes are severely dwarfed (Brown *et al*., 2009; Wu *et al*., 2009, 2010). In poplar, the IRX9, IRX10 and IRX14 genes have also been duplicated (Ratke *et al*., 2015). It was suggested that one set of these poplar genes shows wide expression patterns including in cells that make primary cell walls, as shown here for Arabidopsis. Ratke *et al*. suggested on the basis of this expression pattern that one set may be involved in xylan synthesis in primary cell walls, as now shown here in Arabidopsis. The differentiation of enzymes responsible for the xylan backbone biosynthesis appears to be a result of their specific expression patterns rather than different specificities of the proteins themselves. The *irx9L* and *irx10L* primary wall xylan phenotype could be rescued by expressing *IRX9* under the *IRX9L* promoter, or *IRX10* under the *IRX10L* promoter, respectively.

XylT activity has been recently biochemically demonstrated for IRX10L and IRX10 (Jensen *et al*., 2014; Urbanowicz *et al*., 2014). It remains unclear why IRX9/9L and IRX14/14L are additionally required for xylan backbone synthesis. IRX9, IRX10 and IRX14 are all required for secondary cell wall xylan synthase activity *in vitro,* suggesting they may form part of a complex (Brown *et al*., 2007, 2009), and evidence for TaIRX14:TaIRX10 interaction has been found in wheat (Zeng *et al*. 2010). The *irx9* growth phenotype has been complemented by expression of IRX9 carrying point mutations in the conserved DXD motif (IPR007577), which is predicted to bind the divalent ion required for catalytic activity (Ren *et al*., 2014). The authors proposed a model in which IRX9 and IRX14 may have a structural rather than a catalytic role, anchoring IRX10 to the Golgi membrane, as IRX10 appears not to harbour a transmembrane domain itself (Ren *et al*., 2014). Such a model, in which IRX9L, IRX10L and IRX14 necessarily act in a complex for primary cell wall xylan synthesis, is supported here by the lack of detectable XylT activity in *irx9L* and in *irx14,* and much reduced activity in *irx10L* in microsomal assays and loss of the primary wall xylan in these single mutants. The residual activity seen in *irx10L* could be a result of some functional redundancy with IRX10, which at least according to the transcriptomic data, is still significantly expressed. Alternatively, it could imply that in the absence of IRX10L, IRX9L and IRX14 together are able to extend xylan oligosaccharides at a much reduced rate.

We identified the GT8 family member, GUX3 as the GlcAT for the primary cell wall xylan. Although previously Rennie *et al*. (2012) were unable to detect GlcAT activity of heterologously expressed GUX3, here we showed that the *gux3* mutant lacked *in vitro* GlcAT activity and the primary cell wall xylan lacked GlcA substitution. The other four GT8 family members appear not to have a role in the substitution of this primary cell wall xylan. The function of GUX4 and GUX5 is still unknown. GUX4 and GUX5 could be responsible for adding GlcA to xylan in a different cell type or alternatively, to a different molecule. Recently, a more distantly related GUX1 homologue, IPUT1, has been shown to add GlcA to glycosylinositolphosphoceramides (GIPCs), a class of glycosylated sphingolipids (Rennie *et al*., 2014).

The primary cell wall xylan modified by GUX3 has a fixed interval of six Xyl residues between [Me]GlcA substitutions, as revealed by the GH30 xylanase digestion. This pattern of glucuronoxylan substitutions stands in contrast with the ones described for the major and minor domains substituted by GUX1 and GUX2 for secondary cell wall (Bromley *et al*., 2013). Like GUX3, GUX1 also substitutes xylan by preferentially adding [Me]GlcA to evenly spaced Xyl residues, but with intervals of 8 or 10 predominating. GUX2, however, has no partiality for even or odd spacing. Recent work proposed that the pattern of xylan substitution and xylan acetylation affects the interaction between xylan and cellulose fibrils (Bromley *et al*., 2013; Busse-Wicher *et al*., 2014). The even nature (six residue spacing) of the [Me]GlcA substitution in this primary cell wall xylan could result in a substituted and unsubstituted surface of a flat two-fold screw xylan ribbon (180° bond angle in the xylan backbone), enabling interactions with different cell wall polymers, such as, but not limited to cellulose. Moreover, altering the side chains by the addition of a pentose might change the nature or ability to interact with other cell wall components.

We were not able to determine definitively the nature of the pentose appended to the GlcA due to the low abundance of the structure, although the NMR data and resistance to enzyme hydrolysis suggest an α-1,2-l-Ara*p* rather than a β-1,2-d-Xyl substitution. This situation would be consistent with linkage analysis of Arabidopsis leaf cell wall fractions which revealed small quantities of Ara ascribed to GAX (Zablackis *et al*., 1995). Interestingly, we found no evidence of L-Ara*f* directly linked to the backbone, unlike the GAX of grasses, or the suggested 2-linked L-Ara*f* on the short arabinogalactan-attached xylan chains suggested by (Tan *et al*., 2013). The structure of the PUX_5_ side chains characterized in this study is reminiscent of some grass primary wall xylans, where the side chain Ara is substituted by a Xyl. Here again, the role of these pentosylated side chains is unknown. However, both the Ara side chains (via an added ferulic acid) in grasses and the GlcA side chains in dicots are implicated in cross-linking to other cell wall components. Hence, the addition of pentosyl substitutions to the side chains in the primary cell wall might be a way to influence the cross-linking of xylan.

Arabidopsis primary cell wall xylan is synthesised by a subset of GTs specifically utilising GUX3 for GlcA addition. This results in a xylan substitution pattern that is distinct from the secondary cell wall counterpart, suggesting that the biosynthesis machinery and the resulting xylan structure adjusts to the specific functional roles of xylan in the plant cell wall. This situation may allow the xylan in the primary cell wall to contribute to a flexible, extensible matrix with weaker xylan interactions and reduced cross-linking compared with the more rigid matrix of the secondary cell wall.

## Experimental Procedures

### Plant material and growth conditions

All T-DNA insertion mutant lines were in the Col-0 ecotype. At1g77130 (GUX3): SALK_009975 (*gux3-1*) and SALK_105880 (*gux3-3*) seeds were obtained from the European Arabidopsis Stock Centre (NASC, Nottingham, UK), and GK-166E01 (*gux3-2*) from the GABI-Kat collection at the University of Bielefeld (Kleinboelting *et al*., 2012). At1g08990 (GUX4): SLAT line isolated from pool 02_24_04 (Tissier *et al*., 1999). At1g54940 (GUX5): SALK_123905 (*gux5-1;* NASC). *gux1–2* (SALK_046841), *gux2-1* (GK_722F09), *irx10L* (GK-179G11), *irx9L* (SALK_037323), *irx14* (SALK_038212), *irx9-2* (SALK_057033), *irx10* (SALK_046368) and *irx14L* (SALK_066961) have been described previously (Brown *et al*., 2007, 2009; Mortimer *et al*., 2010). Seeds were surface sterilised and sown on 0.8% (w/v) agar, 0.5 × Murashige and Skoog salts including vitamins (Sigma, http://www.sigma.com) and sucrose (1% w/v). Following stratification for 48 h at 4°C in the dark, plates were transferred to a growth room (20°C, 100 μmol m^−2^ sec^−1^, 24 h light, 60% humidity). After 2–3 weeks, seedlings were transferred to soil (John Innes No. 1, http://www.gardenhealth.com) under the same growth conditions. Liquid callus cultures were generated and maintained as described in Prime *et al*. (2000). Roots were harvested at 15 days, young stems at 26 days, and mature stems at 6 weeks (with at least 10 fully elongated siliques). Homozygous lines were isolated by PCR and confirmed as transcriptional knockouts by RT-PCR as described previously (Mortimer *et al*., 2010). All experiments were performed on at least three independently harvested sets of plant material, although representative data are shown for clarity.

### Glycosylhydrolases used

GH11A xylanase from *Neocallimastix patriciarum* (Gilbert *et al*., 1992), GH30 endoxylanase from *Bacteroides ovatus,* GH3 β-xylosidase (XS) from *Trichoderma reesei* (AN2359.2), GH115 from *Bacteroides ovatus* (Rogowski *et al*., 2014), a GH27 β-l-arabinopyranosidase from *Streptomyces avermitilis* (Ichinose *et al*., 2009), and a GH51 α-l-arabinofuranosidase from *Pseudomonas cellulosa* (Beylot *et al*., 2001). All hydrolases were used in excess to ensure complete digestion of material. The XS was a kind gift from Novozymes, the GH27 was a kind gift from Satoshi Kaneko, National Food Research Institute, Tsukuba, Japan and all other glycoside hydrolases (GHs) were kind gifts from Dave Bolam and Harry Gilbert, University of Newcastle. The action of the enzymes is described in Figure S1(a).

### Microarray data

WT callus (two biological replicates) was harvested and frozen in liquid N_2_. RNA was extracted as described in Mortimer *et al*. (2013). RNA quality was assessed using a Bioanalyzer (Agilent, http://www.genomics.agilent.com) and hybridised to the ATH1 chip (Affymetrix) by the NASC microarray service (Nottingham University, UK). All data are freely available to download at NASC Arrays (http://affymetrix.arabidopsis.info/; Experiment number 704). Data were analysed using the FlexArray software (Blazejczyk *et al*., 2007), and normalised using the Microarray Suite 5 method (MAS5.0) algorithm (Affymetrix 2002). Genes with a two-3fold log change from the WT and a *P*-value < 0.05 and were considered significant. Additional microarray and proteomics data were obtained from publicly available datasets as described in the text.

### Glycosyltransferase (GT) assays

X_6_ (20 μg, Megazyme) was derivatised with 8-aminopyrene-1,3,6-trisulfonic acid (APTS, Biotium) as described in Li *et al*. (2013). Excess fluorophore was removed using a GlykoClean S Cartridges (Prozyme, http://www.prozyme.com) and dried *in vacuo*. The total reaction volume was 60 μl: 30 μl of callus microsomes isolated according to (Mortimer *et al*., 2010) plus 30 μl of 2× reaction buffer. The final reaction conditions were 58 μm derivatised X_6_, 0.25 mm DTT, 5 mm MnCl_2_, 5 mm MgCl_2_, 1% (v/v) Triton X-100 (4-(1,1,3,3-tetramethylbutyl)phenyl-polyethylene glycol), 5 mm UDP-GlcA, 0.16 mm UDP-Xyl in 50 mm HEPES-KOH (pH 7.4). Following incubation for 5 h at 21°C with agitation (50 rpm), the reactions were stopped by heating (100°C, 10 min). To remove lipids a phase separation was used based on (Bligh and Dyer, 1959). To each sample, CHCl_3_:MeOH 1:2 (v/v; 450 μl) was added, followed by 150 μl of CHCl_3_ and 150 μl of H_2_O. The samples were extensively vortexed between each addition. Finally, the samples were centrifuged at 160 ***g*** for 10 min at 21°C. The aqueous phase was collected and dried under vacuum.

### DNA-sequencer assisted saccharide high-throughput analysis (DASH)

Oligosaccharide standards (Megazyme, http://www.megazyme.com) were derivatised with APTS as described above. Assay products, alongside appropriate controls and standards were separated by capillary electrophoresis (CE) according to Li *et al*. (2013). Data was analysed using the DASHboard software (Li *et al*., 2013).

### Alcohol insoluble residue (AIR) preparation

AIR from stems, roots, hypocotyls and callus was prepared as previously described (Goubet *et al*., 2009). Tissue samples were incubated in 96% (v/v) ethanol for 30 min at 70°C. All further steps were performed at room temperature (RT). The material was powdered using a ball mixer (Glen Creston), exchanged twice into 100% (v/v) ethanol, and incubated overnight in chloroform:methanol (2:1). Following a second chloroform:methanol (2:1) wash, the pellet was washed with the following ethanol series: 65% (v/v), 80% (v/v), 100% (v/v) and 100% (v/v), and air dried.

### Enzymatic hydrolysis

AIR (50 μg of mature stem, 200 μg of young stem or 250 μg of root), was pre-treated with 4 m NaOH for 1 h (20 μl, RT). Following neutralisation with HCl, samples were exhaustively digested overnight in 500 μl 0.1 m ammonium acetate (pH 6.0, room temperature, with agitation) using GHs as described in the figure legends and text.

### Polysaccharide analysis by carbohydrate gel electrophoresis (PACE)

GH products, alongside appropriate controls and oligosaccharide standards (Megazyme) were reductively aminated with 8-aminonaphthalene-1,3,6-trisulfonic acid (ANTS; Invitrogen, http://www.lifetechnologies.com), and separated by acrylamide gel electrophoresis according to (Goubet *et al*., 2002). Gels were visualised with a G-box equipped with a short pass detection filter (500–600 nm) and long-wave UV tubes (365 nm emission). Representative gels are shown; experiments were performed on three independently grown biological replicates.

### Immunofluorescence

Sections were prepared and blocked according to Handford *et al*. (2003). Sections were incubated overnight with LM11 (anti-xylan) and stained with Calcofluor White to detect cellulose.

### Matrix-assisted laser desorption/ionization time of flight mass spectrometry (MALDI-ToF-MS)

Enzymatically hydrolysed AIR samples (with or without SEC) were desalted by dialysis (as described above), lyophilised and then further desalted by HyperSep Hypercarb cartridges (Thermo-Fisher, http://www.thermofisher.com). The purified oligosaccharides were reductively aminated with 2-aminobenzoic acid (2-AA; Sigma) using optimised labelling conditions described previously (Ridlova *et al*., 2008). Capillary HILIC was carried out using an LC-Packings Ultimate system (Dionex, http://www.dionex.com) equipped with an amide-80 column (300 μm × 25 cm; 3 μm particle size; Dionex) as previously described (Tryfona *et al*., 2012).

### Size exclusion chromatography (SEC)

AIR (300 mg), hydrolysed with an excess of enzyme (GH11 and GH115), was prepared as described above, desalted by dialysis (MWCO 100–500 Da Spectra/Por, Spectrum Laboratories Inc., http://www.spectrumlabs.com) and lyophilised. Samples were resuspended in 2 ml dH_2_O, and loaded onto a gravity-driven preparative Biogel P2 column (190 × 2.5 cm; Bio-Rad), equilibrated and run in 20 mm ammonium acetate pH 6.0 as previously described (Tryfona *et al*., 2014). Fractions were collected and lyophilised, and the fraction choice was determined by DASH.

### Solution-state NMR

Following SEC, lyophilised samples were resuspended in D_2_O (700 μl; 99.9% purity; Cambridge Isotope Laboratories, http://www.isotope.com) and transferred to a 5-mm NMR tube. NMR spectra were recorded at 298 K with a Bruker AVANCE III spectrometer operating at 600 MHz equipped with a TCI CryoProbe. Two-dimensional ^1^H-^1^H TOCSY, ROESY, ^13^C HSQC and H2BC experiments were performed, using established methods (Cavanagh *et al*., 1995; Nyberg *et al*., 2005); the mixing times were 70 and 200 msec for the TOCSY and ROESY experiments, respectively. Chemical shifts were measured relative to internal acetone (δH = 2.225, δC = 31.07 ppm). Data were processed using the Azara suite of programs (v. 2.8, copyright 1993–2015, Wayne Boucher and Department of Biochemistry, University of Cambridge) and chemical-shift assignment was performed using Analysis v2.4 (Vranken *et al*., 2005).

### Promoter exchanges

The cloning was performed in a pGreenII0000 vector containing the Oleosin–GFP from pFAST-G01 as selection marker (Shimada *et al*., 2010). The IRX9-L and IRX10-L promoter region including the 5′UTR were cloned 1000 bp and 1607 bp upstream of their respective ATG. The promoters were inserted between the *Apa*I at the 5′ and *Eco*RI/*Sal*I at the 3′. The cDNA coding regions of IRX9 and IRX10 were cloned between the *Sal*I at the 5′ and *Cla*I/*Pst*I at the 3′. The stop codons were removed in order to C-terminally fuse in-frame a repeat of four myc tags and a NOS terminator. The tag and terminator were inserted between the *Pst*I at the 5′ and *Sac*I at the 3′. All primers used in this study are described in Table S2.

## SUPPORTING INFORMATION

**Figure S1.** Detection of primary wall xylan.

**Figure S2.** MS (native) analysis of GH30 xylanase hydrolysis of root AIR.

**Figure S3.** GUX genes, mutants and protein identities.

**Figure S4.** PACE fingerprint of xylan structure in *gux* mutant roots.

**Figure S5.** Characterisation of the *gux1gux2gux3* mutant.

**Figure S6.** Expression pattern of xylan-related GTs during root development compared to mature stem, and percent contribution of each member of a subset e.g. GUX was calculated.

**Table S1.** Chemical-shift assignments from NMR.

**Table S2.** Primers used in this study.

**Data S1.** Transcriptomic analysis of WT Col0 Arabidopsis callus using Affymetrix ATH1 chips.
