# Supplementary figures and images for: An unusual xylan in Arabidopsis primary cell walls is synthesised by GUX3, IRX9L, IRX10L and IRX14
Source: Plant J. 2015 Jun 4;83(3):413–26. doi: 10.1111/tpj.12898 (PMC4528235; doi:10.1111/tpj.12898)

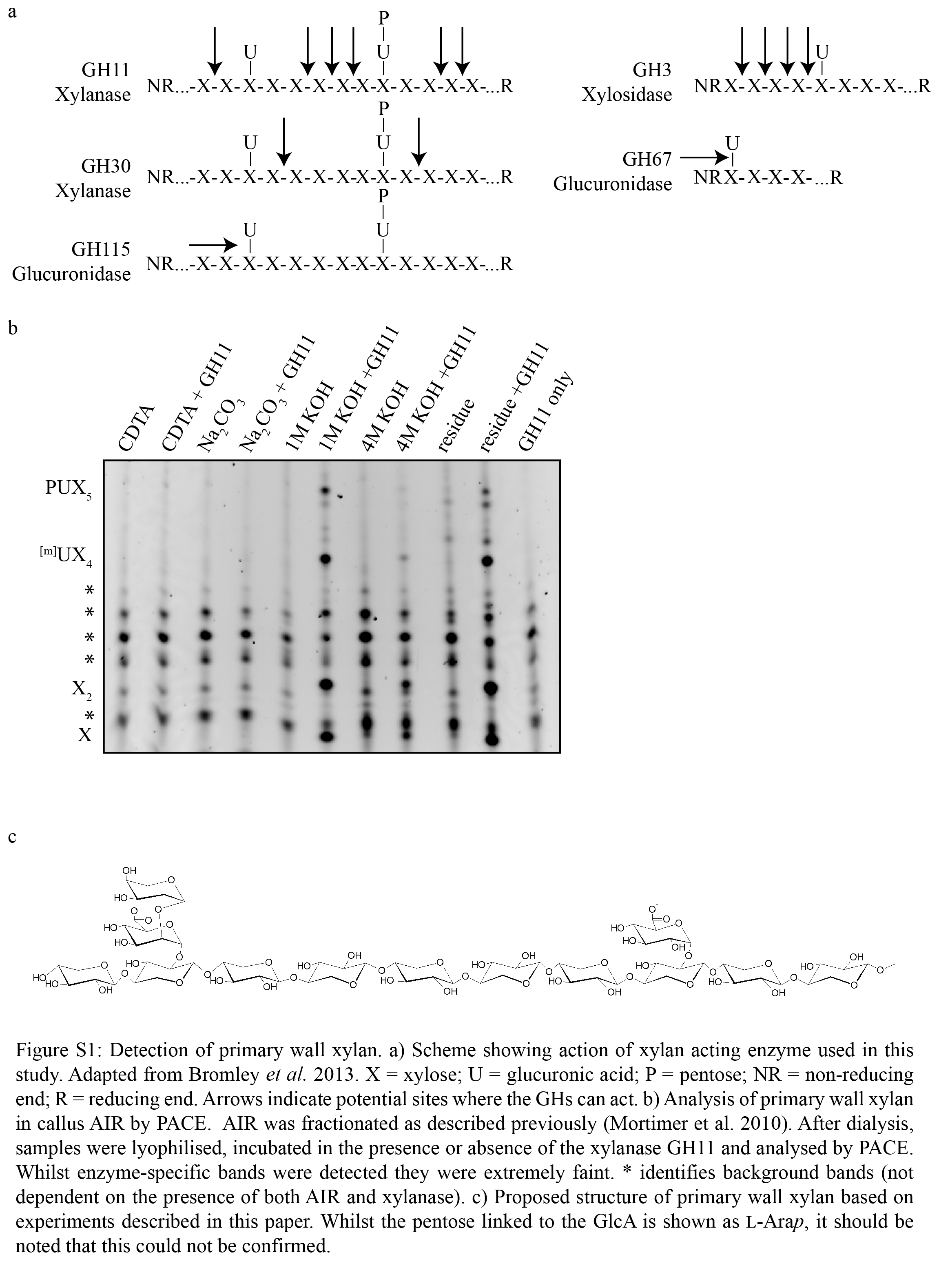

Supplement: Supplementary file 1 — Figure S1. Detection of primary wall xylan. [file tpj0083-0413-sd1.tif]

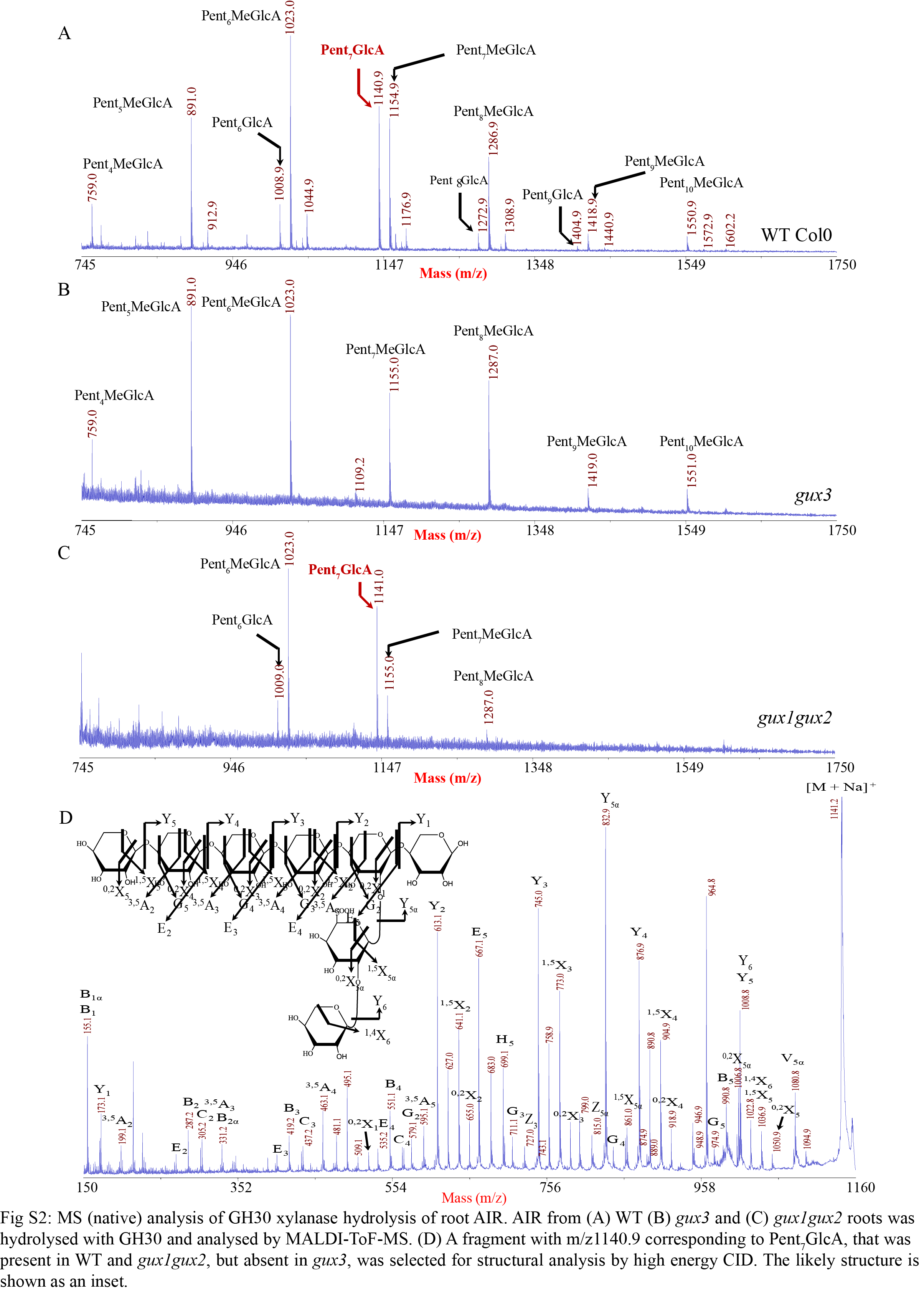

Supplement: Supplementary file 2 — Figure S2. MS (native) analysis of GH30 xylanase hydrolysis of root AIR. [file tpj0083-0413-sd2.tif]

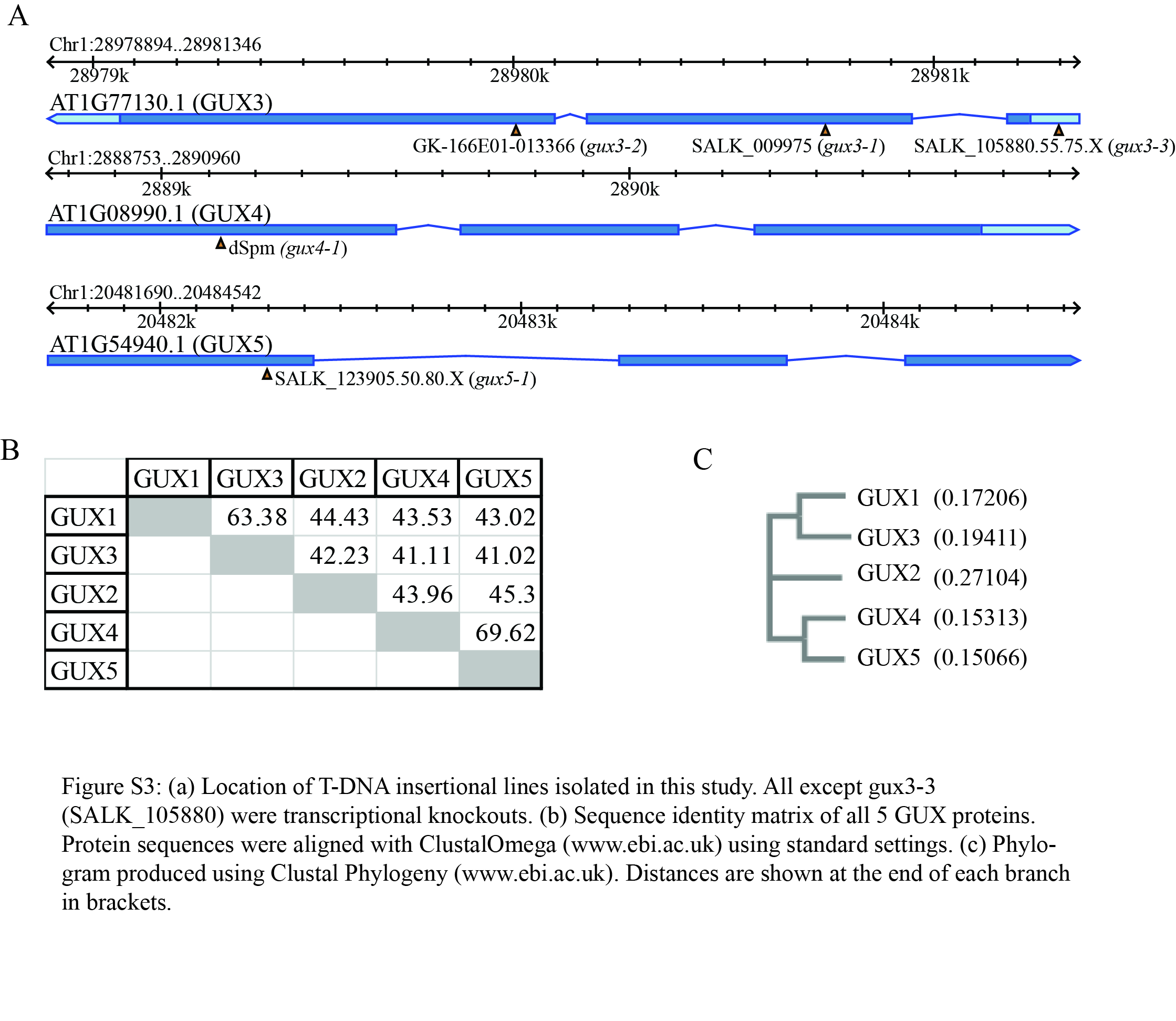

Supplement: Supplementary file 3 — Figure S3. GUX genes, mutants and protein identities. [file tpj0083-0413-sd3.tif]

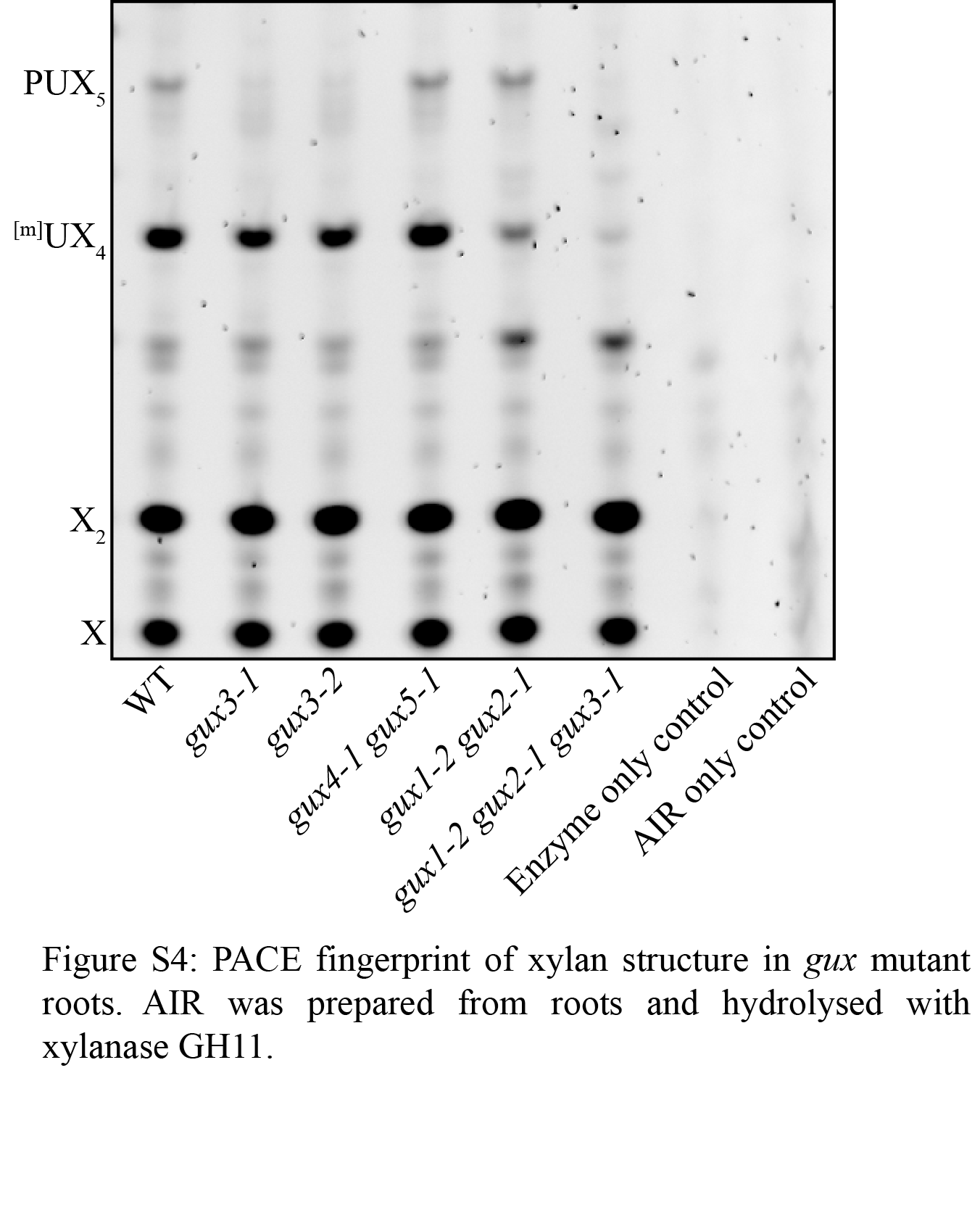

Supplement: Supplementary file 4 — Figure S4. PACE fingerprint of xylan structure in gux mutant roots. [file tpj0083-0413-sd4.tif]

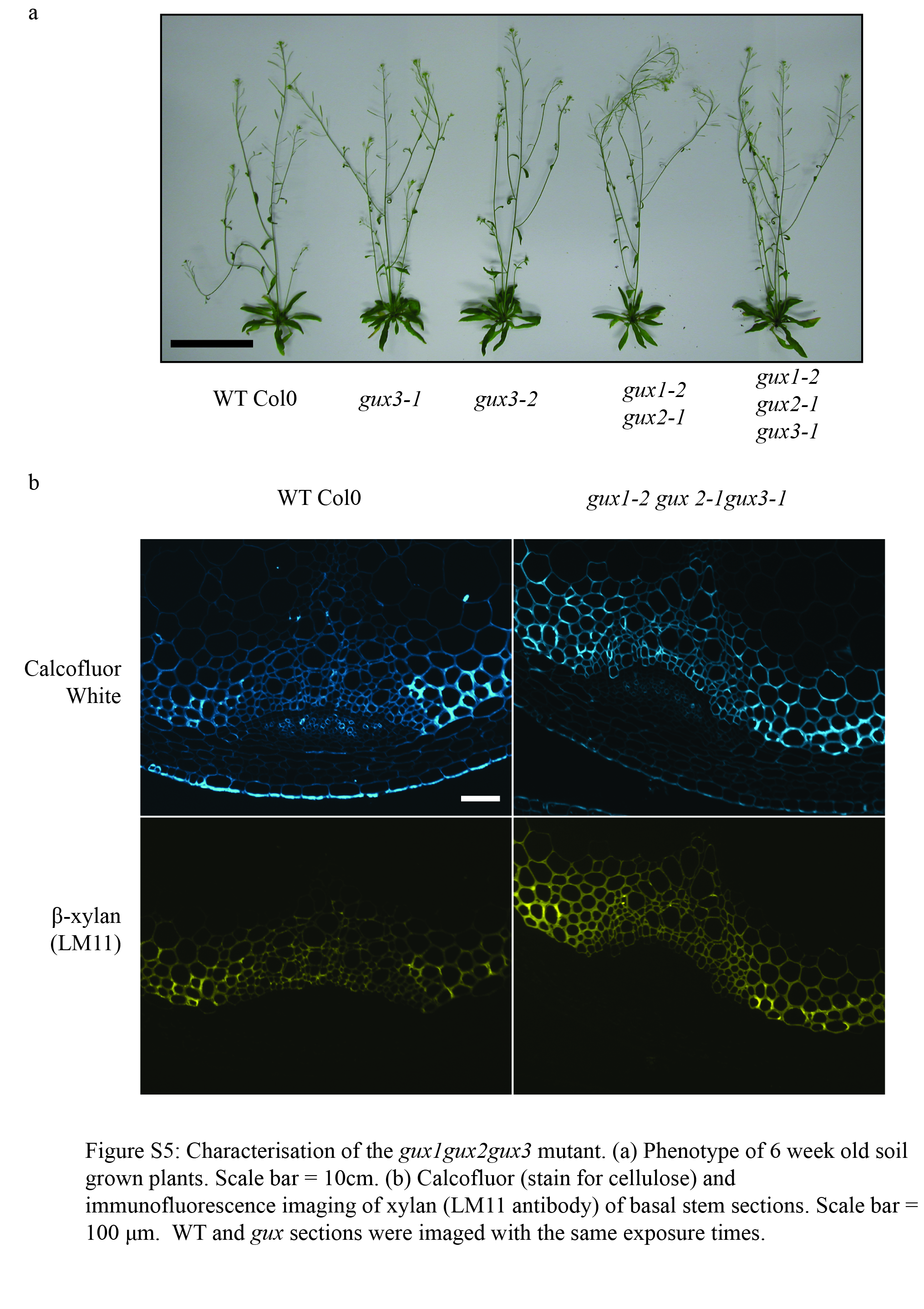

Supplement: Supplementary file 5 — Figure S5. Characterisation of the gux1gux2gux3 mutant. [file tpj0083-0413-sd5.tif]

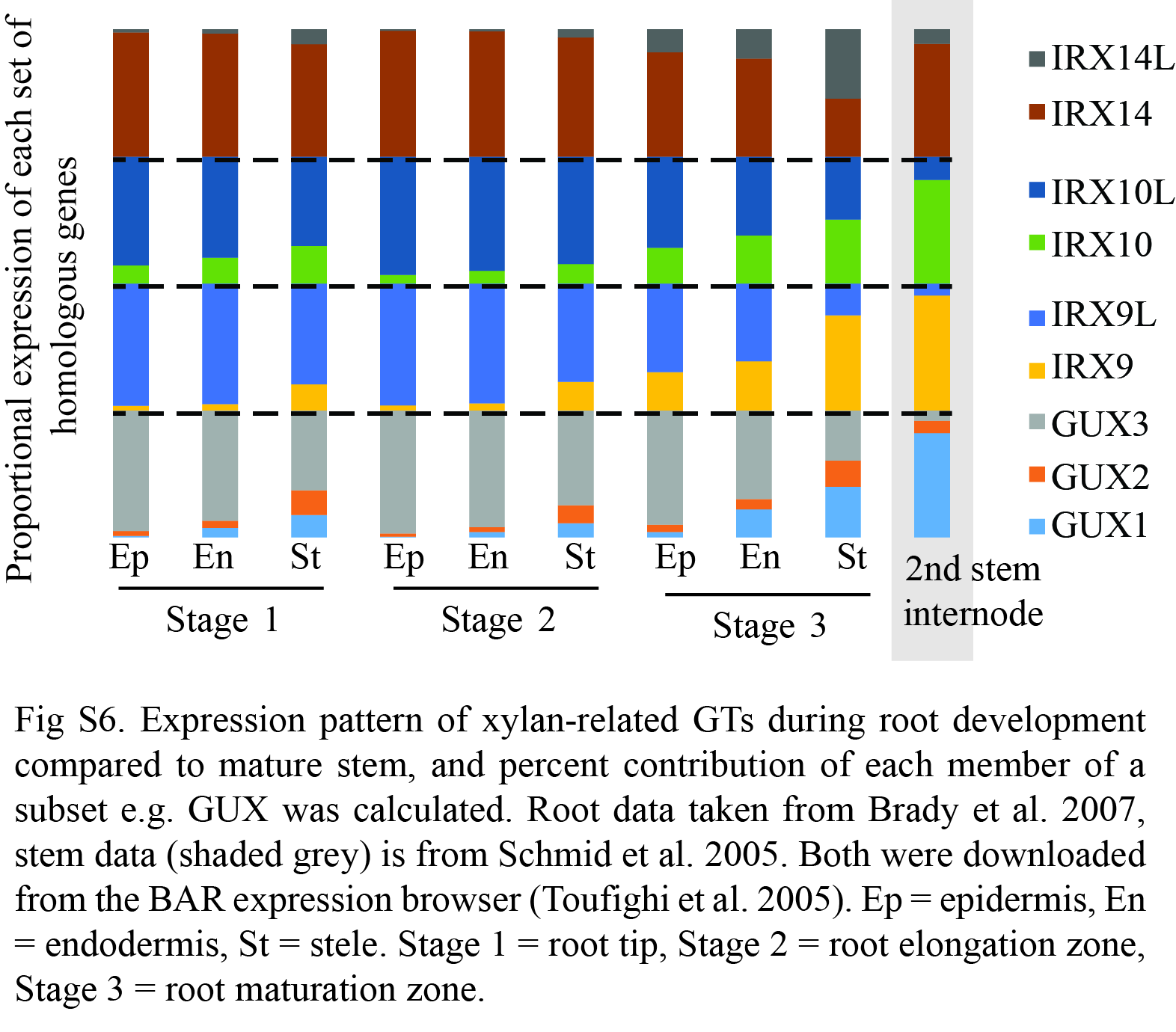

Supplement: Supplementary file 6 — Figure S6. Expression pattern of xylan-related GTs during root development compared to mature stem, and percent contribution of each member of a subset e.g. GUX was calculated. [file tpj0083-0413-sd6.tif]
