# Supplementary material for: An unusual xylan in Arabidopsis primary cell walls is synthesised by GUX3, IRX9L, IRX10L and IRX14
Source: Plant J. 2015 Jun 4;83(3):413–26. doi: 10.1111/tpj.12898 (PMC4528235; doi:10.1111/tpj.12898)
Supplement: Supplementary file 7 — Table S1. Chemical-shift assignments from NMR. [file tpj0083-0413-sd7.docx]

**Table S1. ^1^H and ^13^C NMR assignments of PUX_5_ at 25 °C in D_2_O.**

| Residue |  | Assignment | | | | |
| --- | --- | --- | --- | --- | --- | --- |
|  |  | 1 | 2 | 3 | 4 | 5 |
| β-Xyl*p*_re_ | ^1^H | 4.483 | 3.271 | 3.556 | 3.804 | 4.111, 3.387 |
|  | ^13^C | n.d. | 73.62 | 74.62 | n.d. | 63.80 |
| β -Xyl*p* | ^1^H | 4.507 | 3.279 | 3.590 | 3.805 | 4.165, 3.507 |
|  | ^13^C | 102.56 | 73.62 | 74.49 | n.d. | 63.74 |
| β -Xyl*p* | ^1^H | 4.715 | 3.561 | 3.698 | 3.820 | 3.436, 4.132 |
|  | ^13^C | n.d. | 76.49 | 72.67 | 77.49 | 63.25 |
| β -Xyl*p*_nr_ | ^1^H | 4.464 | 3.276 | 3.435 | 3.625 | 3.313, 3.971 |
|  | ^13^C | 102.80 | 73.65 | 76.38 | 69.93 | 65.96 |
| α-Glc*p*A | ^1^H | 5.419 | 3.764 | 3.943 | 3.538 | 4.377 |
|  | ^13^C | 97.53 | 79.61 | 72.81 | 72.74 | 72.83 |
| α-L-Ara*p*(?) | ^1^H | 4.605 | 3.618 | 3.681 | ~3.946 | ~3.669, 3.939 |
|  | ^13^C | 105.59 | 71.92 | 73.17 | ~69.17 | ~67.38 |
