# Supplementary material for: An unusual xylan in Arabidopsis primary cell walls is synthesised by GUX3, IRX9L, IRX10L and IRX14
Source: Plant J. 2015 Jun 4;83(3):413–26. doi: 10.1111/tpj.12898 (PMC4528235; doi:10.1111/tpj.12898)
Supplement: Supplementary file 8 — Table S2. Primers used in this study. [file tpj0083-0413-sd8.docx]

**Table S2: Primers used in this study**. All primers reported 3’-5’. T-DNA LB primers used were as described for each collection (SALK, SAIL etc).

| **T-DNA insertion lines genotyping and transcript levels.** | |
| --- | --- |
| IRX9 F | TCG TCT AGT ACA TGC GCG ACT TG |
| IRX9 R | ACT CAC TCA AGA AAC CTT CTT CC |
| IRX10 F | CCA CTC GGA GGA CTT GGA |
| IRX10 R | GGA AAA AGC CAT TGA AAG AGG |
| IRX14 F | TGC CTC AAA GCT TGA AGT CTC |
| IRX14 R | AAC GAC ACG TGT ACC TCC TTG |
| IRX9L F | ACT CTT GCA TTC AGG GGA GG |
| IRX9L R | AAA TAG GAC GGT GGA GTG AG |
| IRX10L F | TCA TCC ACA AAC ACA CCA ATA TCT |
| IRX10L R | TTC CAA TTG GCC ATACTGGAA TCG |
| IRX14L F | GTC GTC GAC TCA GCT AGT TGC TGA CAC |
| IRX14L R | GTC CAA TTG AGG ACT GTG ATT GTG GTG |
| GUX1 F | ACA CAA CTG ATA TTC ACC TAA TCT CG |
| GUX1 R | TAA TCT TGA GAA ACA TCG ATT TCT TG |
| GUX2 F | AAA GTG CTA ATT AAT CAC CCT TGA TC |
| GUX2 R | GTG AAA TTG ATG TGA AAG ATA CCA AC |
| GUX3 F | TCCTCA AGT CCC ATG GAG TCA |
| GUX3 R | TGG TGG TGG AAG AAT TGT TGG |
| GUX4 F | ACGTCG TCG TTG TCA AAG TCC |
| GUX4 R | TGA GCT TAG CCA TTT TCC TCC A |
| GUX5 F | AAA TGG CTT CGA CTC ATC CGT |
| GUX5 R | TTT TTC GTG CAC AGA ATC GCT |
| HISTONE1 F | GGC TGC TCC GGT TAA GAA GAA |
| HISTONE1 R | TTT GGA AAG CAA GCT CCT TCA |
| **IRX PROMOTER SWAP** | |
| ApaI-NatPromIRX9-L-F | ATG CGA GGG CCC TTT GAC ACA AAA ACT TAT GTA AAT CTA TTT TTG A |
| NatPromIRX9-L-  EcoRI/SalI-R | CGC CAT GTC GAC GAA TTC CAG ATA ATC TCA CTC CAC CGT CCT ATT TTT CAC AGC TCC CAA |
| ApaI-NatPromIRX10-L-F | ATG CGA GGG CCC CCA TAA TTC CAT ATA TGT CTA TCT |
| NatPromIRX10-L-  EcoRI/SalI-R | GCC ATG TCG ACG AAT TCT TTT CTC TCT CAG AAA TTT TGG TT |
| EcoRI/SalI-IRX10-F | GAA TTC GTC GAC ATG AAA ATC CAC TCT TGC CTC TCC GCC A |
| IRX10-ClaI/PstI-R | CTG CAG ATC GAT CCA AGG TTT CAG GTC AGC AAC CGG ACC AGC A |
| EcoRI/SalI-IRX9-F | GAA TTC GTC GAC ATG GGA TCT CTA GAG AGA TCA AAG AAG AAA GCT CAA |
| IRX9-ClaI/PstI-R | CTG CAG ATC GAT GGT GCT TAA ACG TGT TCT TGTG GGA AAT TTG AGA CGC  CA |
