# Supplementary material for: An unusual xylan in Arabidopsis primary cell walls is synthesised by GUX3, IRX9L, IRX10L and IRX14
Source: Plant J. 2015 Jun 4;83(3):413–26. doi: 10.1111/tpj.12898 (PMC4528235; doi:10.1111/tpj.12898)
Supplement: Supplementary file 10 — Data S1. Transcriptomic analysis of WT Col0 Arabidopsis callus using Affymetrix ATH1 chips. [file tpj0083-0413-sd10.docx]

**Supplemental Data Legends for Mortimer et al.**

**Supplemental Dataset 1**: Transcriptomic analysis of WT Col0 Arabidopsis callus using Affymetrix ATH1 chips.

**Supplemental Tables**

**Supplemental Table 1:** Chemical shift assignments from NMR

**Supplemental Table 2:** Primers used in this study for PCR and RT-PCR. All sequences are reported 3’-5’.

**Supplemental Figures**

**Figure S1:** Detection of primary wall xylan. a) Scheme showing action of xylan acting enzyme used in this study. Adapted from Bromley *et al.* 2013. X = xylose; U = glucuronic acid; P = pentose. Arrows indicate potential sites where the GHs can act. b) Analysis of primary wall xylan in callus AIR by PACE. AIR was fractionated as described previously (Mortimer *et al.* 2010). After dialysis, samples were lyophilised, incubated in the presence or absence of the xylanase GH11 and analysed by PACE. Whilst enzyme-specific bands were detected they were extremely faint. * identifies background bands (not dependent on the presence of both AIR and xylanase). c) Proposed structure of primary wall xylan based on experiments described in this paper. Whilst the pentose linked to the GlcA is shown as Ara, it should be noted that this could not be confirmed.

**Figure S2:** MS (native) analysis of GH30 xylanase hydrolysis of root AIR. AIR from (a) WT (b) *gux3* and (c) *gux1gux2* roots was hydrolysed with GH30 and analysed by MALDI-ToF-MS. (d) A fragment with *m/z* 1140.9 corresponding to Pent_7_GlcA, that was present in WT and *gux1gux2*, but absent in *gux*3, was selected for structural analysis by high energy CID. The likely structure is shown.

**Figure S3**: GUX genes, mutants and protein identities. (a) Location of T-DNA insertional lines isolated in this study. All except *gux3-3* (SALK_105880) were transcriptional knockouts. (b) Sequence identity matrix of all 5 GUX proteins. Protein sequences were aligned with ClustalOmega (www.ebi.ac.uk) using standard settings. (c) Phylogram produced using Clustal Phylogeny (www.ebi.ac.uk). Distances are shown at the end of each branch in brackets.

**Figure S4**: PACE fingerprint of xylan structure in *gux* mutant roots. AIR was prepared from roots and hydrolysed with xylanase GH11.

**Figure S5**: Characterisation of the *gux1gux2gux3* mutant. (a) Phenotype of 6 week old soil grown plants. Scale bar = 10cm. (b) Calcofluor (stain for cellulose) and immunofluorescence imaging of xylan (LM11 antibody) of basal stem sections. Scale bar = 100 μm. WT and *gux* sections were imaged with the same exposure times.

**Figure S6**: Expression pattern of xylan-related GTs during root development compared to mature stem, and percent contribution of each member of a subset e.g. GUX was calculated. Root data taken from ([Brady et al., 2007](#_ENREF_5)), stem data (shaded grey) is from ([Schmid et al., 2005](#_ENREF_41)). Both were downloaded from the BAR expression browser ([Toufighi et al., 2005](#_ENREF_44)). Ep = epidermis, En = endodermis, St = stele. Stage 1 = root tip, Stage 2 = root elongation zone, Stage 3 = root maturation zone.
